# Self-reported assessment of female sexual function among Brazilian undergraduate healthcare students: a cross-sectional study (survey)

**DOI:** 10.1590/1516-3180.2018.0005240418

**Published:** 2018-08-13

**Authors:** Juliana Tamy Satake, Thalita Rodrigues Christovam Pereira, Mariana Chaves Aveiro

**Affiliations:** I BSc. Physiotherapist and Specialist in Women’s Health, Universidade Estadual de Campinas (UNICAMP), Campinas (SP), Brazil.; II MSc. Physiotherapist, Postgraduate Program on Interdisciplinary Health Sciences, Universidade Federal de São Paulo (UNIFESP), Santos (SP), Brazil.; III PhD. Assistant Professor IV, Department of Human Movement Sciences, Universidade Federal de São Paulo (UNIFESP), Santos (SP), Brazil.

**Keywords:** Sexuality, Women’s health, Sexual dysfunctions, psychological, Physical therapy modalities

## Abstract

**BACKGROUND:**

The present study aimed to evaluate female sexual function among young undergraduate women.

**DESIGN AND SETTING:**

Cross-sectional survey conducted among Brazilian undergraduate students.

**METHODS:**

This study used online questionnaires to assess sociodemographic and health-related data and used the Brazilian version of the Female Sexual Function Index (FSFI) among female undergraduate students aged 18 to 25 years who were regularly enrolled in undergraduate healthcare courses. The FSFI is composed of 19 items that measure female sexual function over the last four weeks, in six domains: desire and subjective stimulation, sexual arousal, lubrication, orgasm, satisfaction and pain or discomfort.

**RESULTS:**

Among the 149 female undergraduate students evaluated, 43 (28.8%) presented sexual dysfunction (score < 26.55). Health conditions were not associated with female sexual dysfunction. Among the women with sexual dysfunction, all domains of the sexual response cycle were affected (P < 0.001).

**CONCLUSIONS:**

Sexual dysfunction was identified in at least a quarter of these young undergraduate women and it was not associated with gynecological problems, menstrual cycles, dysmenorrhea, contraceptive use or physical activity.

## INTRODUCTION

Female sexuality was historically treated as taboo in some cultures and was deemed to be restricted to procreation and distant from pleasure. Today, women’s sexuality is considered to be an integral part of their sexual rights and quality of life that is important not only for reproduction but also for longevity of their affective and pleasurable relationships, as well as being part of their health and wellbeing.^1^

Sexual function and dysfunction present multifactorial characteristics that lead to a range of psychological, interpersonal, sociocultural and neurobiological factors.^2^ Female sexual dysfunction encompasses a wide variety of clinical conditions, including hypoactive sexual desire, sexual aversion disorder, sexual arousal disorder, orgasmic disorder and painful disorders such as dyspareunia and vaginismus.^3^

Although female sexual response has not been completely elucidated, it is known that female sexual function involves somatic, psychosocial and neurobiological factors.^4^ Any disturbance or change in sexual function, such as pain and discomfort during sexual intercourse, can compromise women’s wellbeing and quality of life.

The World Health Organization (WHO) recognizes female sexual dysfunction as a public health problem and recommends that it should be investigated in the event of important changes in quality of life.^5^ Impaired female sexual function (problems with sexual desire, arousal, orgasm and sexual pain) causes high levels of personal or interpersonal distress.^6^

The majority of Brazilian studies on female sexual function have investigated this among women who had some disease or were in a specific reproductive period, such as pregnancy or the menopause.^7^ Studies that evaluate female sexual function among Brazilian students are scarce, but it is known that the prevalence of sexual dysfunction increases with age and multiparity and after the menopause.^3^

There are differences in female sexual function relating to the demographic variables of different countries and between individuals, such that these affect individuals’ behavior and the sexual practices that they adopt. Thus, the prevalence of sexual dysfunction varies. Therefore, evaluations on sexuality should not be generalized for the entire female population but should have a specific focus for each population studied.^5^^,^^8^ It is important to know about the different female sexual responses within different populations, like young undergraduate women.

Hence, because of the scarcity of research addressing groups of young women in Brazil, the aim of the present study was to evaluate female sexual function among young undergraduate women.

## METHODS

### Study design, date, setting and ethical issues

This was a cross-sectional survey conducted on the Baixada Santista campus of the Federal University of São Paulo (Universidade Federal de São Paulo), in Santos, state of São Paulo, between August and December 2012. This study was approved by the Ethics Committee for Human Research (under number 32,649/2012) and all the participants provided written informed consent. The “STrengthening the Reporting of OBservational studies in Epidemiology” (STROBE) statement was used for reporting the study.

### Participants

We included female undergraduate students aged 18 to 25 years who were regularly enrolled in some of the undergraduate healthcare courses (physiotherapy, occupational therapy, physical education, psychology, nutrition and social service focusing on healthcare interdisciplinarity) on the Baixada Santista campus, in Santos, were personally contacted and invited to participate in the study. Those who agreed to participate answered the questionnaires online.

We excluded women who had not had sexual intercourse within the previous four weeks, since this is a criterion for responses that are used to form the Female Sexual Function Index (FSFI). We also excluded women who had had children or who were pregnant because these conditions may interfere with sexual function. In addition, women who had never had sexual intercourse were excluded because experience of sexual intercourse is a condition for answering the FSFI questionnaire^5^.

A recent study revealed that almost 40% of undergraduate students were at risk of female sexual dysfunction.^15^ This frequency for the primary outcome was therefore taken as an assumption. The sample size for this study was calculated considering a 95% confidence level and a sample error of 10%. Thus, it was found that the sample size needed to be 93 women.

#### Data collection, variables and analysis

An online questionnaire was sent by e-mail to women with an interest in participating in the study. The information collected through this questionnaire comprised age, undergraduate course, age at menarche, gynecological problems, information on the menstrual cycle (regular or irregular), dysmenorrhea, contraceptives, age at first sexual intercourse and physical activity. The level of physical activity was classified as follows: sedentary (does not perform any physical activity for at least 10 continuous minutes during the week); irregularly active (performs physical activity, but insufficient to be classified as active, as this does not comply with the recommendations regarding frequency or duration); active (vigorous activity ≥ 3 days/week lasting ≥ 20 minutes/session, or moderate activity/walking ≥ 5 days/week lasting ≥ 30 minutes/session, or any added activity ≥ 5 days/week lasting ≥ 150 minutes/week); or very active (vigorous activity ≥ 5 days/week lasting ≥ 30 minutes/session, or vigorous activity ≥ 3 days/week lasting ≥ 20 minutes/session + moderate activity/walking ≥ 3 days/week lasting ≥ 30 minutes/session).^9^

Every participant also answered the online Brazilian version of the Female Sexual Function Index (FSFI) questionnaire, which had been adapted and validated for the purpose of assessing female sexual function.^5^^,^^10^^,^^11^^,^^12^ This is a simple and objective questionnaire composed of 19 items that measure female sexual function over the last four weeks. The FSFI is the only index addressing affective, emotional and psychosocial issues and it includes the following six domains of female sexual response: desire and subjective stimulation, sexual arousal, lubrication, orgasm, satisfaction and pain or discomfort. In addition, this instrument has gone through a process of verification of its trustworthiness and reliability as an online version.^12^

The results from the questions that comprised each domain were multiplied by the factor for that domain. The scores from each domain were summed, resulting in the final FSFI score. The final score was obtained by summing the weighted scores of each domain. The final scores could range from 2 to 36, and higher scores represented better female sexual function. As previously established in the literature,^13^ women with scores less than or equal to 26.55 were considered to have some sexual dysfunction.^5^^,^^8^^,^^10^^,^^11^^,^^12^^,^^14^

### Statistical analysis

The participants’ characteristics were analyzed using descriptive statistics. The chi-square test was used to verify whether there were any associations between presence of sexual dysfunction and the variables of interest. The Mann-Whitney test was used to verify whether there was any difference between women with sexual dysfunction and women without sexual dysfunction, in relation to the variables. The significance level used for every comparison was 0.05 (P ≤ 0.05).

## RESULTS

**Figure 1:**
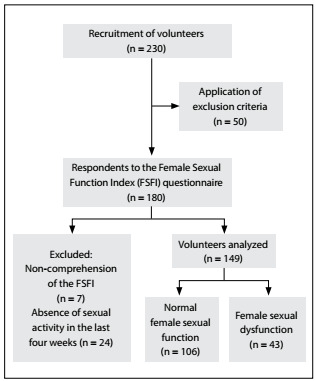
Recruitment and distribution of volunteers.

In total, 230 female undergraduate students were invited to participate, and 180 (78.2%) of these students answered the questionnaires online. Thirty-one were excluded because they had not had sexual intercourse within the last four weeks (Figure 1).^5^ Thus, 149 undergraduate students (Figure 1) from the following healthcare courses were included: physiotherapy, occupational therapy, physical education, psychology, nutrition and social service focusing on healthcare interdisciplinarity. The participants’ average age was 21 years (± 1.68) (Table 1) and, among them, 43 (28.8%) were categorized as having dysfunction because they presented a Female Sexual Function Index of less than 26.55.

**Table 1:** Distribution of the number of volunteers according to course and undergraduate level

Characteristics	n	%
Undergraduate course		
Physiotherapy	25	16.78
Occupational therapy	25	16.78
Physical education	20	13.42
Social service focusing on healthcare interdisciplinarity	27	18.12
Psychology	26	17.45
Nutrition	26	17.45
Undergraduate level		
1^st^ year	22	14.77
2^nd^ year	31	20.81
3^rd^ year	41	27.52
4^th^ year	51	34.23
5^th^ year	4	2.68

The women with sexual dysfunction did not differ from the women without sexual dysfunction in relation to gynecological problems, menstrual cycles, contraceptives, dysmenorrhea or physical activity (Table 2). The age at the menarche was on average 12.13 years (± 1.14) for the women with sexual dysfunction and 12.17 years (± 1.55) for the women without sexual dysfunction (P = 0.922). In relation to the age at the first sexual intercourse, there was also no significant difference between the women with sexual dysfunction (17.02 ± 1.72 years) and those without sexual dysfunction (17.33 ± 2.06 years) (P = 0.485).

**Table 2:** Health conditions among women with and without sexual dysfunction

	With sexual dysfunction		Without sexual dysfunction		P*
	n	%	n	%	
Gynecological problems					
Present	5	11.63	15	14.15	0.68
Absent	38	88.37	91	85.85	
Menstrual cycle					
Regular	36	83.72	86	81.13	0.71
Irregular	7	16.28	20	18.87	
Dysmenorrhea					
Present	27	62.79	67	63.20	0.96
Absent	16	37.21	39	36.80	
Contraceptives					
Yes	33	76.74	73	68.87	0.34
No	10	23.26	33	31.13	
Physical activity					
Sedentary	5	11.63	20	18.87	0.26
Irregularly active	9	20.93	32	30.19	
Active	21	48.84	43	40.57	
Very active	8	18.60	11	10.38	

n = sample number; % = frequency (percentage); *chi-square test with significance level of P < 0.05.

**Figure 2:**
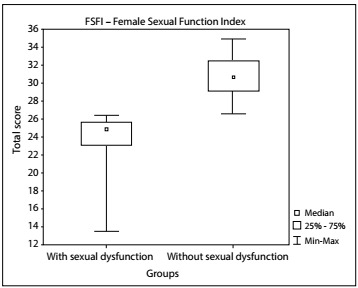
Comparison of the categories of with and without sexual dysfunction through the Brazilian version of the Female Sexual Function Index (FSFI) (P ≤ 0.001; Mann-Whitney test).

The total FSFI score (Figure 2) and the scores for each domain of the FSFI were compared among the women, both with and without sexual dysfunction. Among those with sexual dysfunction, it could be seen that all the FSFI domains were affected (Table 3).

**Table 3:** Domains of Female Sexual Function Index among women with and without sexual dysfunction

Domains of Female Sexual Function Index	With sexual dysfunction		Without sexual dysfunction		P*
	Median	Interquartile range	Median	Interquartile range	
Desire	3.6	3-3.6	4.2	3.6-4.8	< 0.001
Arousal	4.2	3.6-4.5	5.4	5.1-5.7	< 0.001
Lubrication	4.8	3.9-5.1	5.4	5.1-6	< 0.001
Orgasm	3.6	2.8-4.4	5.2	4.4-5.6	< 0.001
Satisfaction	4.8	3.6-5.2	5.6	5.2-6	< 0.001
Pain	4.4	3.6-5.4	5.2	4.8-6	< 0.001
Total score	24.9	23.3-25.6	30.7	29.2-32.5	< 0.001

*Mann-Whitney test with significance level of P < 0.05.

## DISCUSSION

Sexual dysfunction was found in 28.8% of the undergraduate students who participated in this study, with a mean age of 20.9 years old. Moreover, every domain was affected among the women with sexual dysfunction, i.e. orgasm, desire, arousal, pain, lubrication and satisfaction.

Although there was an increase in interest in publishing data on female sexual function in Brazil between 2013 and 2015,^7^ gaps in the literature still exist,^3^ especially with regard to young women. A recent Brazilian systematic review^7^ pointed out that few articles had good methodology and used validated questionnaires to assess sexual dysfunction among women. Among the articles included in that systematic review, the majority related to female sexual function among patients with some type of disease or investigated sexual function at specific reproductive periods, such as during pregnancy or at the menopause.

Young women, such as undergraduate students, are often considered healthy and do not show any impairment of their sexual health. Taking into account that the age at the first sexual intercourse in the present study was on average 17 years, the women included in this survey were at the beginning of their sexual life. Sexual dysfunction during this period affects the quality of life of young and healthy women.

In a recent study^15^ involving female medical students in German-speaking countries with a mean age of 23.5 years, it was revealed that almost 40% were at risk of female sexual dysfunction. It was demonstrated that being in a steady relationship, having better physical fitness, being more active at work and having greater subjective positive self-acceptance were associated with higher total scores in the Female Sexual Function Index. Thus, individuals with these characteristics were at lower risk of presenting female sexual dysfunction.

Considering that the prevalence of sexual dysfunction increases with age and parity and after the menopause, investigation of the quality of sexual health among undergraduate students is necessary in order to support preventive action, health promotion and functional recuperation designed specifically for young Brazilian women.

The literature indicates that lubrication dysfunction is more often observed during the climacteric period, due to the various hormonal changes that occur in this period.^15^ Another important point to be emphasized is that absence of sexual dysfunction does not necessarily imply sexual satisfaction.^2^

Regarding the health conditions investigated among the volunteers of the present study, it seems that there were no differences between the women with sexual dysfunction and the women without sexual dysfunction. However, further studies should be conducted to investigate the association of sexual dysfunction with health conditions throughout female sexual life. Recently, some studies have demonstrated the existence of associations between sexual dysfunction and other health conditions such as pubic pain,^17^ type II diabetes^18^ and conditions subsequent to conventional abdominal hysterectomy.^19^ Another point to be discussed is that the present study assessed self-reported sexual function among young women through the Female Sexual Function Index. This questionnaire is quick and easy to apply and has high reliability for diagnosing the presence or absence of sexual dysfunction subjectively, without presenting the causes of this disorder. Thus, it would be of interest to carry out a physical evaluation in order to complement the Female Sexual Function Index results and to identify possible physical causes of sexual dysfunction.

Unfortunately, there is a scarcity of studies evaluating the presence of sexual dysfunction in populations of young and healthy women in Brazil. Most studies in the literature relate to women in the climacteric or gestational period, or to women who presented some form of disorder or chronic disease.^7^^,^^16^^,^^20^^,^^21^^,^^22^^,^^23^

Higgins et al.^24^ investigated the physiological and psychological satisfaction with sexual life among American undergraduate students. They found that several of the same individual, relationship and cultural-level factors correlated with sexual satisfaction among adults, regardless of gender. However, they highlighted some differences between the genders. For example, men were twice as likely as women to report that they always or almost always experienced an orgasm during sexual intercourse.

Brazilian conservative society still sees female orgasm as unnecessary while male orgasm is synonymous with virility. In this regard, many women no longer resort to healthcare services in order to improve their sexual health because of the belief that lack of orgasmic experiences is inherent to the female organism.

The present study was not intended to differentiate women according to sexual orientation. Perhaps this is a limitation of the study, since the Brazilian version of the Female Sexual Function Index does not report the partner’s sex as an inclusion criterion.^5^^,^^10^^,^^11^^,^^12^ Therefore, new studies should be designed to provide validated instruments capable of assessing female sexual function regardless of sexual orientation.

Treatments for sexual dysfunction are essentially transdisciplinary and include not only collaboration among multidisciplinary professionals but also application of collaborative practice to ensure the highest level of provision of care.^2^ For the physical aspects of sexual dysfunction, physiotherapy may be beneficial.

Among the possibilities for physiotherapeutic intervention, studies have shown the effectiveness of raising awareness of the pelvic floor muscles and strengthening them, which alters female sexual life positively. Furthermore, some studies have shown that awareness and proprioception of the musculature improve self-image, receptivity towards sexual activity and sexual performance.^25^ Use of electrostimulation with biofeedback has also proved to be an effective technique for treating sexual dysfunctions, through increased learning and control of pelvic floor muscle contraction. This, together with regard for the contraction force provided by biofeedback, aids in improvement of performance and reduces pain.^26^

Thus, early diagnosing of female sexual dysfunction in young women, along with transdisciplinary intervention, may contribute towards improvement of sexual practice and quality of life. This approach may provide new possibilities for treatment for this younger population.

## CONCLUSION

Sexual dysfunction was identified in at least a quarter of the young undergraduate women surveyed. It was not associated with gynecological problems, menstrual cycles, dysmenorrhea, contraceptive use or physical activity.
